# Fine‐needle aspiration of parathyroid adenomas: Indications as a diagnostic approach

**DOI:** 10.1002/dc.24595

**Published:** 2020-08-24

**Authors:** Ayana Suzuki, Mitsuyoshi Hirokawa, Risa Kanematsu, Aki Tanaka, Naoki Yamao, Miyoko Higuchi, Toshitetsu Hayashi, Seiji Kuma, Akihiro Miya, Akira Miyauchi

**Affiliations:** ^1^ Department of Diagnostic Pathology and Cytology Kuma Hospital Kobe Japan; ^2^ Department of Surgery Kuma Hospital Kobe Japan

**Keywords:** cytology, fine‐needle aspiration, GATA‐3, parathyroid adenoma, parathyroid hormone

## Abstract

**Background:**

We aimed to determine the indication of fine‐needle aspiration (FNA) for parathyroid adenoma (PA)‐suspected nodules and the cytological features of PA, and to discuss the ancillary techniques for diagnostic confirmation.

**Method:**

Clinical, cytological, and histological examinations of 15 PA patients (4.0% of all PA resected patients) were conducted through FNA on 16 nodules. We also examined the cytological preparations of 10 follicular neoplasms (FNs) and 10 poorly differentiated thyroid carcinomas (PDTCs).

**Results:**

FNA was performed to detect PA in nine (56.3%) nodules. The remaining seven (43.8%) nodules underwent FNA for lesions considered as thyroid nodules or lymph nodes. The levels of parathyroid hormone (PTH) in the aspiration needle washout fluid were observably high, except for that from one nodule with unsatisfactory FNA. Cytologically, the incidences of wedge pattern (86.7%) and salt and pepper chromatin (86.7%) in PAs were significantly higher than in FNs and PDTCs. In contrast, the appearance of colloid globules and nuclear grooves was less frequent than that of FNs and PDTCs. GATA‐3 expression was intense in all PAs that immunocytochemistry were performed. Histologically, capsular invasion and/or laceration, tumor seeding, granulation tissue, and fibrosis were observed.

**Conclusions:**

When PA localization is unusual or inconclusive despite extensive imaging, FNA may be performed. We asserted that wedge pattern, salt and pepper chromatin, and the absence of colloid globules and nuclear grooves are diagnostic cytological indicators of PA rather than of FN or PDTC. We recommend PTH measurements using needle washout fluid for PA‐suspected nodules, and immunocytochemistry with the GATA‐3 antibody for cytologically PA‐suspected nodules.

## 1 INTRODUCTION

Parathyroid adenoma (PA) is the most common cause of primary hyperparathyroidism, and the first‐line therapy involves the surgical resection of the tumor.^1^ To identify the localization of PA in patients with primary hyperparathyroidism at a preoperative stage, three examinations: ultrasound examination (UE), ^99m^Tc‐MIBI scintigraphy, and computerized tomography with enhancement, are generally performed, and these diagnostic modalities achieve the diagnostic goal in almost all cases.^2^, ^3^ Fine‐needle aspiration (FNA), generally considered a useful preoperative diagnostic tool for tumors, is not recommended for PA‐suspected tumors owing to the chances of serious complications, such as massive hematoma, parathyromatosis, and misdiagnosis as malignancy during pathological diagnosis.^2^, ^3^ However, when the localization is unusual or PA is mistaken for a thyroid nodule, FNA may be indicated or performed erroneously.^4^, ^5^

There are limited articles in existing literature related to FNA in PA, and the diagnostic accuracy is relatively low.^1^, ^4^ PAs are frequently misdiagnosed as follicular neoplasms (FNs).^6^ The suitability of FNA in PA should be discussed with caution. This study aimed to determine the indication of FNA for PA‐suspected nodules and the cytological features of PA, and to discuss ancillary techniques for PA diagnosis.

## 2 MATERIALS AND METHODS

We studied 374 patients with PA who underwent resection at the Kuma Hospital between January 2016 and December 2018. Among them, 15 patients (4.0%) underwent FNA for 16 PA nodules. FNA was performed using a 22‐gauge needle under ultrasound guidance. The aspirated materials were prepared by the press and release method,^7^ and were immediately fixed with Cytorop (Alfresa Pharma Co., Osaka, Japan), a cytological fixative. The samples were subsequently stained using the Papanicolaou method. After smearing, the syringe and needle were rinsed with 0.5 mL saline, and the levels of parathyroid hormone (PTH) were measured in the washout fluid from 10 out of 16 PA nodules (62.5%). Clinical data and ultrasonography reports were collected from the medical records of Kuma Hospital. The standard values of serum calcium and intact PTH (i‐PTH) levels were determined to be 8.2 to 10.2 mg/dL and 15 to 70 pg/mL, respectively.

Among the 16 PAs on which FNA was performed, 15 were studied cytologically. The sixteenth sample was excluded owing to poor cellularity. We also examined the preparations of 10 FNs (8 adenomas and 2 carcinomas) and 10 poorly differentiated thyroid carcinomas (PDTCs) that were aspirated during the same period. Table 1 shows the definitions of the main cytological findings used in this study. Immunocytochemical examination was performed using the following primary antibodies: GATA‐3 (L50‐823, Biocare Medical, Concord, California), chromogranin A (DAK‐A3, Dako, Glostrup, Denmark), PTH (105G7, Novocastra, Newcastle, UK), paired‐box gene 8 (PAX8) (EPR13510, Abcam, Cambridge, UK), and thyroid transcription factor‐1 (TTF‐1) (8G7G3/1, Dako, Carpinteria, California). Staining was performed using the Leica Bondmax system and Bond refine kit (Leica Microsystems, Wetzlar, Germany) according to the manufacturer's recommendations. The histological preparations of 16 PAs were evaluated with respect to the effect of preoperative FNA.

**TABLE 1:** Definitions of the main cytological findings used in this study

Colloid globules	Rounded proteinaceous material measuring 5 to 100 μm in diameter
Capillaries	Capillaries embedded within the large cell cluster or naked capillaries in the background
Tissue fragments	Lump composed of tumor cells and stromal components
Trabecular pattern	Slender cell cluster showing two or more rows
Wedge pattern	Triangular cell cluster with one sharp corner
Insular pattern	Large and solid cell cluster without stromal components
Cribriform pattern	Large cell cluster including some microfollicular or slit‐like lumens
Microfollicular pattern	Follicular or glandular cell cluster composed of less than 15 tumor cells
Round cell predominant	More than 50% of tumor cells exhibiting completely round nuclei
Anisonucleosis	Nuclei with variability of more than twice in size
Prominent nucleolus	The size of nucleolus is more than 20% of its nucleus

We determined the statistical significance of data using Fisher's exact probability test. *P* value <.05 was considered to be statistically significant.

## 3 RESULTS

### 3.1 Clinical findings

The median age of the patients was 61 years (range 41‐71 years). The female‐to‐male ratio was 12:3. Serum calcium levels were measured prior to thyroid FNA in all cases, and ranged from 9.7 to 12.8 mg/dL (median: 10.8). In 12 patients (80%), the serum calcium levels were higher than normal values. The levels of i‐PTH ranged from 76 to 1537 pg/mL (median: 149 pg/mL) and were elevated in all samples.

Based on the UE reports, seven (43.8%) and six (37.5%) nodules were considered to be intrathyroid and extrathyroid lesions, respectively. The location of the remaining three nodules was not determined to be intrathyroidal or extrathyroidal. Out of the 16 nodules, 8 (50.0%), 7 (43.8%), and 1 (6.3%) were suspected to be PA, thyroid nodules, and lymph nodes, respectively.

### 3.2 FNA

FNA was performed to determine the location of PA in nine (56.3%) nodules collected from patients with primary hyperparathyroidism. In the remaining seven (43.8%) nodules, FNA was performed for lesions erroneously recognized as thyroid nodules or lymph nodes in UE. In eight (88.9%) of the former and five (71.4%) of the latter, PA was correctly interpreted, with or without immunocytochemical analysis. One nodule (6.3%) was considered of unsatisfactory quality owing to poor cellularity. Differential diagnoses included FN (37.5%), benign follicular lesions (18.8%), and PDTC (12.5%). FNA‐induced hematomas or parathyromatosis were not clinically observed.

### 3.3 Cytological findings

Table 2 outlines the cytological findings of 15 PAs, 10 FNs, and 10 PDTCs.

**TABLE 2:** Cytological findings from 15 parathyroid adenomas, 10 follicular thyroid neoplasms, and 10 poorly differentiated thyroid carcinomas

	PAs (15)	FNs (10)	PDTCs (10)
*Background*			
Colloid globules	13.3% (2)	70.0% (7)**	80.0% (8)**
Foamy histiocytes	6.7% (1)	0% (0)	10.0% (1)
Neutrophils	0% (0)	0% (0)	0% (0)
Lymphocytes	0% (0)	0% (0)	10.0% (1)
Capillaries	20.0% (3)	60.0% (6)	50.0% (5)
*Arrangements*			
Tissue fragments	60.0% (9)	50.0% (5)	60.0% (6)
Trabecular	93.3% (14)	80.0% (8)	70.0% (7)
Wedge	86.7% (13)	0% (0)***	30.0% (3)**
Insular	80.0% (12)	0% (0)***	50.0% (5)
Cribriform	53.3% (8)	0% (0)***	80.0% (8)
Microfollicular	26.7% (4)	100% (10)**	70.0% (7)
Isolated	53.3% (8)	30.0% (3)	70.0% (7)
Naked cells	73.3% (11)	40.0% (4)	30.0% (3)*
*Tumor cells*			
Oxyphilic cytoplasm	53.3% (8)	30.0% (3)	20.0% (2)
Round cell predominant	100% (15)	90.0% (9)	30.0% (3)***
Multinucleation	0% (0)	30.0% (3)	40.0% (4)*
Anisonucleosis	26.7% (4)	50.0% (5)	40.0% (4)
Nuclear grooves	0% (0)	40.0% (4)*	80.0% (8)***
Salt and pepper chromatin	86.7% (13)	0% (0)***	20.0% (2)**
Prominent nucleoli	0% (0)	20.0% (2)	30.0% (3)

*Note*: Vs parathyroid adenoma.

Abbreviations: FN, follicular neoplasm; PA, parathyroid adenoma; PDTC, poorly differentiated thyroid carcinoma.

* *P* < .05.

** *P* < .01.

*** *P* < .001.

#### 3.3.1 Background

Colloid globules were more frequently observed in FNs (70.0%) and PDTCs (80.0%) than in PAs (13.3%) (*P* < .01, *P* < .01). Entangled capillaries were detected in 20.0%, 60.0%, and 50.0% of PAs, FNs, and PDTCs, respectively. Inflammatory cells were rarely observed in any of the three lesions.

#### 3.3.2 Arrangements

**FIGURE 1:**
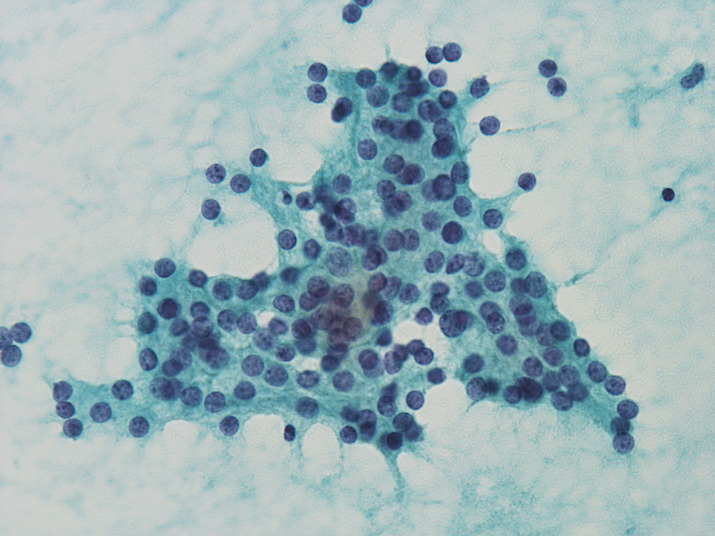
Parathyroid adenoma. The cluster presented here exhibits a wedge pattern (×40, Papanicolaou) [Colour figure can be viewed at wileyonlinelibrary.com]

Tissue fragments composed of tumor cells and stromal components were observed in 60%, 50%, and 60% of PAs, FNs, and PDTCs, respectively. Trabecular arrangement was frequently detected in all tumors. Triangular cell clusters with one sharp corner (wedge pattern) (Figure 1) were observed in 86.7% of PAs, and the incidence was significantly higher than that of FNs (*P* < .001) and PDTCs (*P* < .01). Insular and cribriform patterns were observed in PAs (80.0%, 53.3%) and PDTCs (50.0%, 80.0%), while it was not observed in any of the FNs. While all FNs displayed a microfollicular pattern, the incidence of the pattern was low in PAs (26.7%, *P* < .01). Naked cells were more frequently detected in PAs (73.3%) than in PDTCs (30.0%) (*P* < .05).

#### 3.3.3 Tumor cells

**FIGURE 2:**
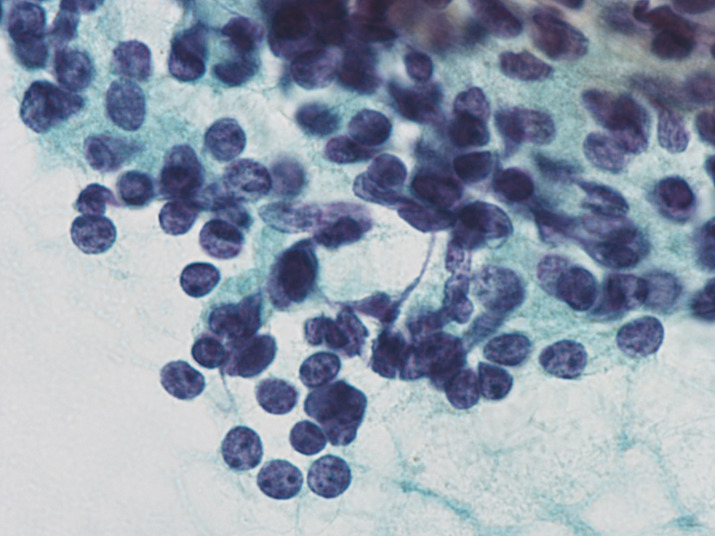
Parathyroid adenoma. The nuclei exhibit salt and pepper chromatin patterns (×100, Papanicolaou) [Colour figure can be viewed at wileyonlinelibrary.com]

Oxyphilic cytoplasm was observed in 53.3% of PAs, while intracytoplasmic fat vacuoles were not detected in all cases of PA. The nuclei in PAs were predominantly rounded in shape and did not exhibit multinucleation or the presence of nuclear grooves or prominent nucleoli. The incidence of salt and pepper chromatin pattern (Figure 2) was significantly higher in PAs (86.7%) than in FNs (0%, *P* < .001) or PDTCs (20.0%, *P* < .01).

### 3.4 PTH assay

The levels of parathyroid hormone (PTH) were measured in the aspiration needle washout fluid from 10 out of 16 PA nodules (62.5%). The levels of i‐PTH and highly sensitive PTH (HS‐PTH) were measured in two and seven nodules, respectively. In the remaining nodule, both parameters were measured. i‐PTH and HS‐PTH levels ranged from 355 pg/mL to 5000 pg/mL and from 211 pg/mL to 2 700 000 pg/mL, respectively. The levels were observably higher than the normal ranges in serum (i‐PTH: 15‐70 pg/mL, HS‐PTH: 74‐273 pg/mL), except for that in one nodule in which FNA was deemed unsatisfactory.

### 3.5 Immunocytochemical analysis

Immunocytochemical staining was performed in five PAs, since PA was suspected based on cytological findings. GATA‐3 was detected in all PAs. PTH and chromogranin A were examine in three and two PAs, with positive rates of 66.7% (2/3) and 100% (2/2), respectively. PAX8 was undetected in all three PAs examined. One PA showed the presence of a limited number of TTF‐1‐positive cells, which were considered to be contaminated with thyroid follicular cells.

### 3.6 Pathological findings

**FIGURE 3:**
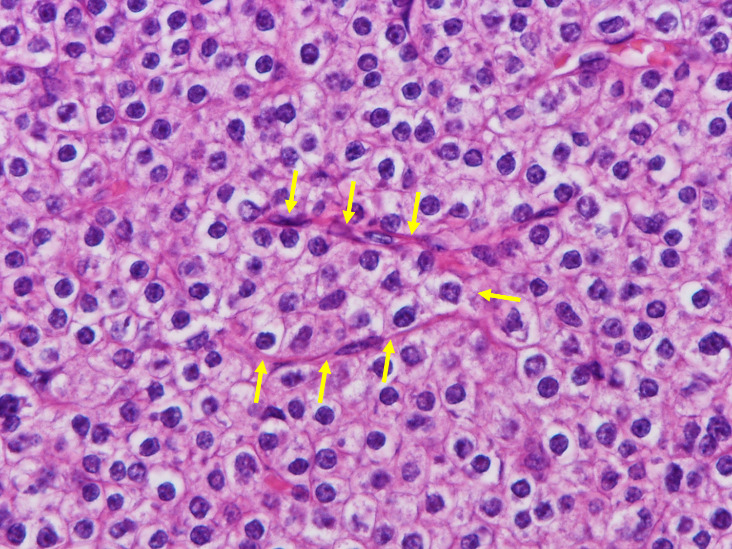
Parathyroid adenoma. Triangular tumor nest surrounded by capillaries (arrows) can be seen (×40, Hematoxylin & Eosin) [Colour figure can be viewed at wileyonlinelibrary.com]

**FIGURE 4:**
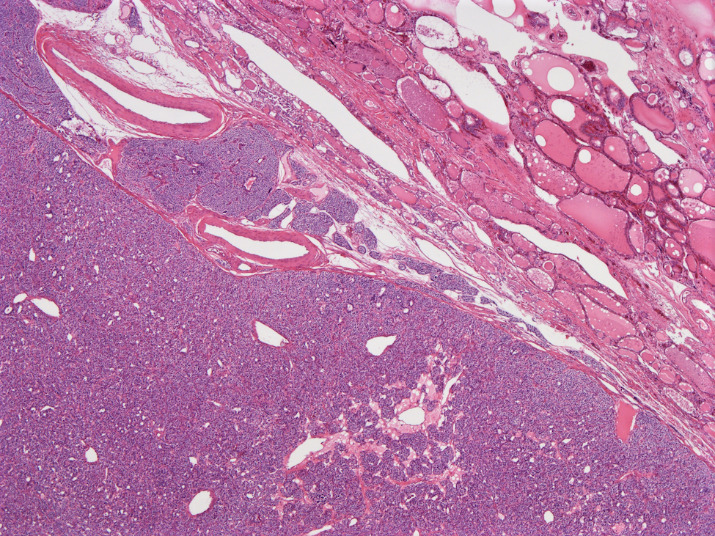
Parathyroid adenoma components present outside of the primary tumor (×10, Hematoxylin & Eosin) [Colour figure can be viewed at wileyonlinelibrary.com]

Out of the 16 PA nodules, 15 were located in the extrathyroid region (upper left: 1, lower left: 5, upper right: 5, and lower right: 4); the remaining PA was located within the right lobe. All PA nodules were composed of neoplastic chief cells. Moreover, tumor cells showed either a solid, alveolar, trabecular, or glandular growth pattern. Noteworthy, triangle tumor nests surrounded by capillaries were scattered in all PAs. (Figure 3). Hemorrhages were not observed on the cut surface. Capsular invasion and/or laceration were observed in 9 (56.3%) out of 16 PAs. One PA (6.3%) that was resected 1157 days after FNA exhibited tumor seeding (Figure 4). Granulation tissue and fibrosis were observed in one (6.3%) and six nodules (37.5%), respectively. Microscopic hemorrhage, hemosiderin deposition, or necrosis were not observed in any of the PAs.

## 4 DISCUSSION

According to the guidelines of the American Association of Endocrine Surgeons, preoperative parathyroid FNA is not recommended, except in unusual and difficult cases of primary hyperparathyroidism, and should not be performed if parathyroid carcinoma is suspected.^8^, ^9^ FNA in parathyroid neoplasms increases the risk of massive hemorrhage, tumor seeding, parathyromatosis, or recurrence.^2^, ^10^, ^11^ The risk is especially high in cases of parathyroid carcinomas.^12^ Bancos et al reported that immediate hematoma occurred in 5% of parathyroid FNA cases, and delayed complications such as inflammatory response, abscess formation, and hematoma were observed in 10% of cases.^3^ The procedure can cause histological changes that mimic parathyroid carcinoma.^8^ However, Knezevic et al and Dimashkieh et al described FNA as a useful diagnostic tool for PA.^13^, ^14^

We opined that FNA should be avoided in the preoperative diagnosis for PA to the maximum extent possible. FNA has been performed on only 4.0% of PA resected patients at our institution. Although the frequency appears remarkably low, this could not be confirmed since there are no related reports. In the current study, only two types of cases were determined to be appropriate for FNA. In 56.3% of cases, the purpose of FNA was to determine the localization of PA‐suspected nodules. In the remaining cases, FNA was performed without suspecting PA. This scenario is not unusual; some extrathyroidal lesions such as esophageal diverticula, paratracheal cysts or parathyroid lesions may be ultrasonically misinterpreted as thyroid lesions.^15^ In fact, the lesions observed in this study are completely extrathyroidal or incompletely embedded in the thyroid, as 15 of our 16 PA nodules. We believe that both scenarios were appropriate. However, it is notable that 80% of the cases with FNA exhibited hypercalcemia. The erroneous use of FNA could be reduced if the requisite attention is provided during examination.

Fortunately, clinical complications such as hematoma, parathyromatosis, and recurrence were not observed in the cases studied here. The primary purpose of FNA for PA‐suspected nodules is the measurement of PTH levels in the needle washout fluid. Therefore, in such cases, we ensured that the needle was not moved considerably during the aspiration. This might have helped reduce the risk of complications. In resected preparations, capsular invasion, capsular laceration, tumor seeding, granulation, and fibrosis were observed. These findings may be attributed to FNA.^16^, ^17^ When we histologically examine PAs with a history of FNA, it is necessary to consider its effect.

Knezevic‐Obad et al reported that the inadequate rate of parathyroid FNA (40%) was significantly higher than that of thyroid nodules (12%), owing to the depth of location and intensive blood circulation.^13^ However, as the inadequate rate in the current study was 6.3%, the inadequacy of parathyroid FNA was not proved. According to the previous reports, the diagnostic accuracy of FNA in PAs ranged from 72% to 80%.^1^, ^5^ Heo et al reported that the accuracy of FNA for PA‐suspected nodules (86.7%) was considerably higher than that for unsuspected nodules (50.0%).^1^ Clearly, the cytological diagnosis of PA was influenced by clinical information. Similarly, in our study, the accuracy for PA‐suspected nodules tended to be higher than that for unsuspected nodules. However, the difference was not statistically significant. To avoid references to misleading clinical information, we recommend observing the cytological preparations before referring to clinical information, as we practiced.

The cytological findings of PAs have been described in several reports.^1^, ^4^, ^5^, ^13^, ^14^, ^17^, ^18^, ^19^, ^20^, ^21^, ^22^ Generally, colloid globules, foamy histiocytes, and capillaries were present in 12% to 53%, 0% to 29%, and 73.3% of PAs, respectively.^1^, ^4^, ^14^, ^19^, ^21^ In our study, the incidences of colloid globules (13.3%) and foamy histiocytes (6.7%) were similar, whereas capillaries appeared in only 20.0% of PAs. Microfollicular pattern and naked nuclei have been described as cellular arrangements of PA.^1^, ^4^, ^14^, ^18^, ^21^ However, the wedge pattern should be examined more carefully than the microfollicular pattern. The wedge pattern was observed in 86.7% of PAs. Thus far, this finding has not been described with respect to PAs; however, we believe it to be a characteristic pattern of PAs and is likely derived from triangular tumor nests surrounded by capillaries that were observed in histological preparations. Intracytoplasmic fat vacuoles may appear in Giemsa‐stained samples^19^; however, we could not observe the findings because they are almost invisible in wet‐fixed Papanicolaou‐stained preparations.^22^, ^23^ Oxyphilic cytoplasm has been observed in 50% of the PAs.^1^, ^5^, ^19^, ^21^, ^22^ The incidence was almost the same in the cases studied here. Round nuclei, coarsely granular chromatin, and anisonucleosis are characteristic nuclear findings in PAs.^1^, ^4^, ^18^, ^19^ We defined anisonucleosis as the presence of nuclei with variability of more than twice in size. Based on this criteria, anisonucleosis was not observed in approximately three‐fourths of the samples in our study. Therefore, the difference in incidence may depend on the definition.

Cytologically, PAs have often been misdiagnosed as FNs or benign follicular lesions.^1^, ^4^, ^13^ Although a few reports have described the cytological differences between PAs and thyroid follicular cells,^4^, ^18^, ^19^ the distinction between PA and follicular cells was not sufficiently clear. In addition, we also needed to differentiate the PDTCs in 12.5% of PAs. PA can be composed of oxyphilic tumor cells, but there were no cases that needed to be distinguished from the oxyphilic variant of follicular neoplasm or papillary carcinoma in the present study. This study is the first to compare the cytological observations of PA, FN, and PDTC samples in detail. Capillaries, naked cells, and anisonucleosis have been reported as typical characteristics of PAs, although we observed them in the FNs and PDTCs in our study. We believe that these findings are not important in differential diagnosis. Certain studies had reported that PAs can easily be confused with papillary thyroid carcinoma^13^, ^20^; however, there were no such cases in our study.

In this study, wedge, insular, and cribriform patterns and salt and pepper chromatin were more frequently observed in PAs than in FNs. In contrast, colloid globules and nuclear grooves were less frequent in PAs. Compared to PDTCs, wedge pattern, naked cells, predominant round nuclei, and salt and pepper chromatin were more frequent in PAs, whereas colloid globules, multinucleation, and nuclear grooves were less frequent. Therefore, the cell arrangements of PAs were similar to those of PDTCs, whereas the nuclear observations of PAs were similar to those of FNs. In conclusion, wedge pattern, salt and pepper chromatin, and the absence of colloid globules and nuclear grooves are better diagnostic indicators of PA than of FN or PDTC. Among the findings, the wedge pattern is a novel indicator proposed by us.

When the localization of PA‐suspected nodules is unusual or inconclusive despite extensive imaging, FNA may be indicated. In such cases, measurement of PTH levels in the needle washout fluid is particularly effective and the sensitivities (87.0%‐93.6%) and specificities (91.2%‐100%) are considerably high.^22^, ^24^, ^25^, ^26^ In our study, the sensitivity of PTH measurement was 90.0%. One false‐negative case was attributed to aspiration failure. It is necessary to understand that the reliability of PTH measurement depends on the aspiration procedure. According to previous reports,^27^, ^28^, ^29^ i‐PTH, HS‐PTH, and C‐PTH levels are generally measured and have been found to be useful as indicators that confirm the presence of parathyroid lesions. However, i‐PTH level measurement is not recommended for parathyroid cysts because PTH is rapidly degraded into the biologically inactive C‐terminal peptide in the cyst fluid.^27^

Immunocytochemistry also serves as a useful tool for determining the parathyroid derived cells in cytological preparations. To determine the status of the parathyroid gland, immunocytochemistry is usually performed using PTH antibodies.^5^, ^14^, ^21^, ^22^, ^30^ However, PTH immunostaining occasionally yields negative results and cannot be used for naked cells owing to its cytoplasmic localization.^31^ In the current study, one out of the three PAs in which PTH immunostaining was performed yielded negative staining results because almost all PA cells appeared as naked nuclei. Conversely, GATA‐3 immunostaining showed intense nuclear positivity, with a sensitivity of 100%. We recommend intense nuclear positivity in immunostaining with the GATA‐3 antibody as an indicator instead of PTH measurement for distinguishing between parathyroid cells and thyroid follicular cells.

Certain reports suggest that molecular testing programs, such as the Afirma gene expression classifier and ThyroSeq, are useful for parathyroid lesions detection^5^, ^18^, ^21^; however, in Japan, molecular testing of aspirated materials is not performed because it is expensive and not covered by medical insurance.

In conclusion, when the localization of PA‐suspected nodules is unusual or inconclusive even when extensive imaging has been performed, FNA may be recommended. In such cases, we perform FNA in a short period of time to avoid complications. To reduce the frequency of FNA for ultrasonically intrathyroidal unknown FAs, serum calcium levels should be checked before FNA. In addition, upon the examination of histological preparations of PA, the histological changes caused by preoperative FNA should be considered. As the cytological findings of PA overlap with those of FN or PDTC, we should always consider including PA for the differential diagnosis of FN or PDTC. The wedge pattern, salt and pepper chromatin, and the absence of colloid globules and nuclear grooves are diagnostic indicators that suggest the presence of PA than that of FN or PDTC. We recommend PTH measurement using needle washout fluid for PA‐suspected nodules, and immunocytochemistry with the GATA‐3 antibody for cytologically PA‐suspected nodules.

## CONFLICT OF INTEREST

The authors declare no conflict of interests associated with this article.

## AUTHOR CONTRIBUTIONS

Akira Miyauchi involved in organization, trial administration, and writing final article. Mitsuyoshi Hirokawa involved in trial coordination and administration, and writing final article. Ayana Suzuki involved in chief investigator, data analysis, trial administration, and writing final article. Risa Kanematsu, Aki Tanaka, Naoki Yamao, Miyoko Higuchi, Toshitetsu Hayashi, Seiji Kuma, and Akihiro Miya involved in trial design, trial administration, and writing final article.
